# Thermodynamics Explains How Solution Composition Affects the Kinetics of Stochastic Ice Nucleation

**DOI:** 10.1021/acs.jpclett.3c01371

**Published:** 2023-06-22

**Authors:** Leif-Thore Deck, Lisanne Wittenberg, Marco Mazzotti

**Affiliations:** Institute of Energy and Process Engineering, ETH Zurich, 8092 Zurich, Switzerland

## Abstract

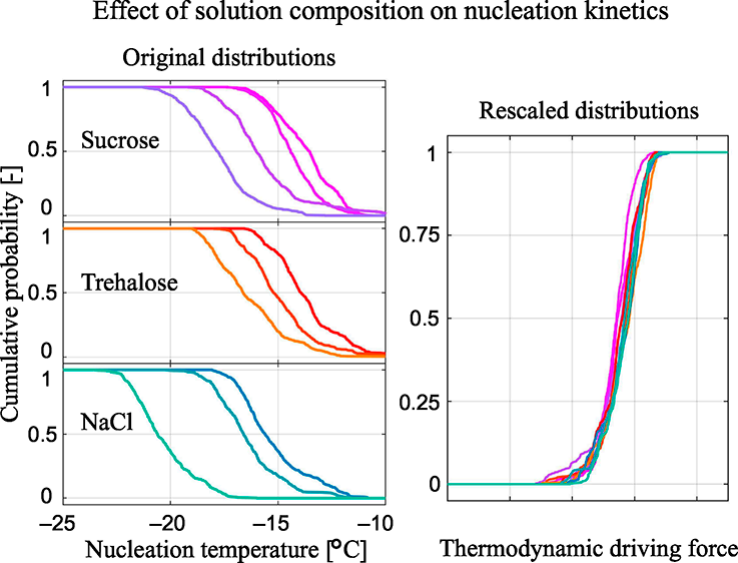

The freezing of aqueous solutions is of great relevance to multiple fields, yet the kinetics of ice nucleation, its first step, remains poorly understood. The literature focuses on the freezing of microdroplets, and it is unclear if those findings can be generalized and extended to larger volumes such as those used in the freezing of biopharmaceuticals. To this end, we study ice nucleation from aqueous solutions of ten different compositions in vials at the milliliter scale. The statistical analysis of the approximately 6,000 measured nucleation events reveals that the stochastic ice nucleation kinetics is independent of the nature and concentration of the solute. We demonstrate this by estimating the values of the kinetic parameters in the nucleation rate expression for the selected solution compositions, and we find that a single set of parameters can describe quantitatively the nucleation behavior in all solutions. This holds regardless of whether the nucleation rate is expressed as a function of the chemical potential difference, of the water activity difference, or of the supercooling. While the chemical potential difference is the thermodynamically correct driving force for nucleation and hence is more accurate from a theoretical point of view, the other two expressions allow for an easier implementation in mechanistic freezing models in pharmaceutical manufacturing.

Despite a long history of research on the freezing of aqueous solutions, the nature of some underlying phenomena remains elusive. This is especially true for ice nucleation, which denotes the formation of the first ice crystal from a clear solution.^1−3^ Its kinetics is of great relevance to multiple fields, from cloud microphysics,^4−6^ to cryobiology,^7,8^ to pharmaceutical manufacturing.^2,9^ The formation of a nucleus is a stochastic event,^10,11^ so that solutions of identical composition that are stored under identical conditions nucleate at different, randomly distributed times.^12,13^ Such behavior is highly undesirable in pharmaceutical manufacturing because of its strict quality control regulations for the freezing of biopharmaceutical drug products.^14,15^ The majority of biopharmaceuticals, including most commercially available vaccines against COVID-19, is formulated and distributed in a frozen or freeze-dried state in vials of milliliter scale.^16−18^ Still, mechanistic descriptions of ice nucleation in models for the freezing of these products have become available only recently.^13,17,19^

This is largely because research on ice nucleation has traditionally been driven by the atmospheric sciences.^20−23^ To mimic the properties of cloud droplets, freezing experiments have been predominantly carried out in microdroplets.^6,24−26^ In such small volumes, ice nucleation may occur homogeneously, i.e., independent of the so-called heterogeneous nucleation sites;^6,20^ this is because these sites are located for instance on dust particles, whose concentration is low enough that a sufficiently small droplet contains none of them. For the freezing of biopharmaceuticals in vials, however, nucleation has been shown to be governed by heterogeneous nucleation sites.^19,27,28^ Heterogeneous nucleation has been studied at the microscale as well, by inserting ice-nucleation agents such as mineral dust particles in a controlled manner.^29−32^ Such insertion of foreign materials to control the nucleation rate, however, is unlikely to find acceptance in pharmaceutical manufacturing due to product quality considerations.^27,28^ For these reasons, the large body of literature on ice nucleation has received little attention from researchers and practitioners working on the design and optimization of pharmaceutical freezing processes.

In this contribution, we address this gap by studying ice nucleation in aqueous solutions in vials of milliliter scale, that is, under conditions relevant to pharmaceutical manufacturing. We carried out a large experimental campaign comprising about 6,000 freezing events to accurately capture the stochastic nature of ice nucleation by exploiting a methodology developed recently.^19^ In short, vials were filled with 1 mL of solution and were cooled at a constant cooling rate of 0.6 K min^–1^ to a temperature of −25 °C. The time and temperature when ice nucleation occurred were measured by means of thermocouples inserted into each vial. To generate data sets of statistical relevance, three to four experiments were carried out, each comprising 12 freeze–thaw cycles and 15 vials; this yields 540–720 nucleation temperatures for each solution composition (further information is provided in Methodology). Ten different solutions were studied, which contain sucrose, trehalose, and sodium chloride at different concentrations. The three solutes represent commonly used excipients in biopharmaceutical formulations,^9,33^ and their physicochemical properties are sufficiently different to allow contending that the findings presented here are of general relevance. That is, we show first that the solution composition affects the nucleation behavior predominantly through the thermodynamic properties. Second, we demonstrate that the nucleation rate can be expressed with comparable quantitative accuracy through a driving force given in terms of a difference either in chemical potential, in water activity, or in temperature.

Figure 1 shows all the measurements of nucleation temperatures during a cooling ramp for the ten aqueous solutions considered. Each colored symbol (point) gives the fraction of vials (vertical coordinate) already nucleated at the corresponding temperature (horizontal coordinate); obviously, such a fraction increases monotonically as the temperature decreases, until it reaches one at a temperature low enough where all vials have nucleated. The resulting curve is called the empirical cumulative distribution function (the CDF of nucleation temperatures in this case) and can also be calculated using a nucleation model (these are the black solid lines in Figure 1 that will be discussed below). Considering that the nucleation temperature is measured with an accuracy of ± 0.15 K, that the experimental conditions are the same for all points on a curve (same solute concentration and cooling rate), and that the first and the last nucleation temperatures differ in all ten cases by 5–7 K, one can safely conclude that nucleation is the main source of the observed variability.

**Figure 1:**
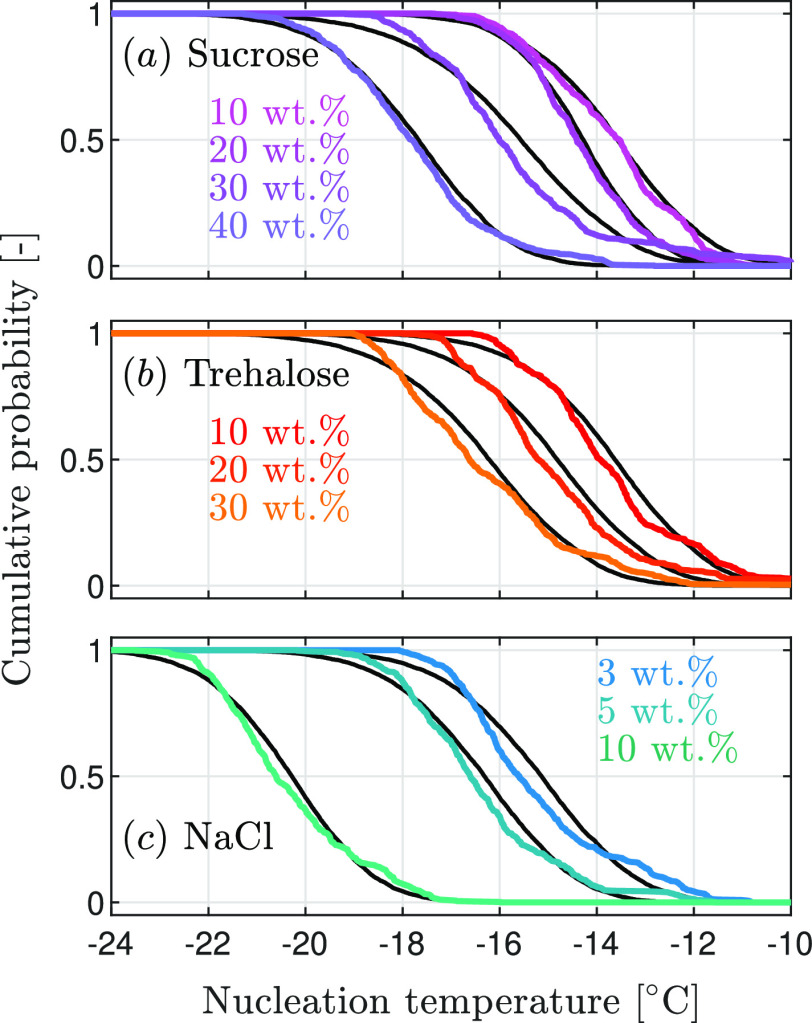
Cumulative distributions of the experimentally measured nucleation temperatures, i.e., the fraction of instances where nucleation has occurred when cooling the solution to a specific temperature. The colored lines represent the experimental data, while the black lines show the optimal model fit for the supercooling-based parametrization of nucleation. (a) Sucrose solutions of four concentration levels. (b) Trehalose solutions of three concentration levels. 40 wt % solutions were not studied due to the lower solubility of trehalose compared to sucrose. (c) Sodium chloride solutions of three concentration levels.

However, Figure 1 does not show *how* nucleation induces this variability, i.e., whether it is due to its inherent stochasticity or to random differences in heterogeneous nucleation sites among vials. In earlier work we have observed that both effects are relevant,^19^ and this is the case here as well. To demonstrate this, we plot in Figure 2 all the 6,000 measured nucleation temperatures. Each panel reports the data for one solution composition; the 12 nucleation temperatures measured per vial are arranged in columns sorted by ascending vial mean nucleation temperature. The horizontal lines indicate the equilibrium freezing temperature, i.e., the melting point. Independent of solution composition, the nucleation temperatures within most vials vary by 2–3 K. As no significant variability among cycles has been observed (not shown explicitly), it is safe to assume that the nucleation sites within each vial remain unchanged throughout an experiment. Hence, the variability in the nucleation temperatures within vials is due to the inherent stochasticity of nucleation. In addition to this inherent stochasticity, the vials with the lowest nucleation temperatures (on the left of each panel) nucleate on average at about 5 K lower temperature levels than those with the highest nucleation temperatures (on the right of each panel). This phenomenon is due to differences in the nucleation sites among vials: the vials that nucleate earlier must contain either more numerous or more active sites than the late-nucleating ones.

**Figure 2:**
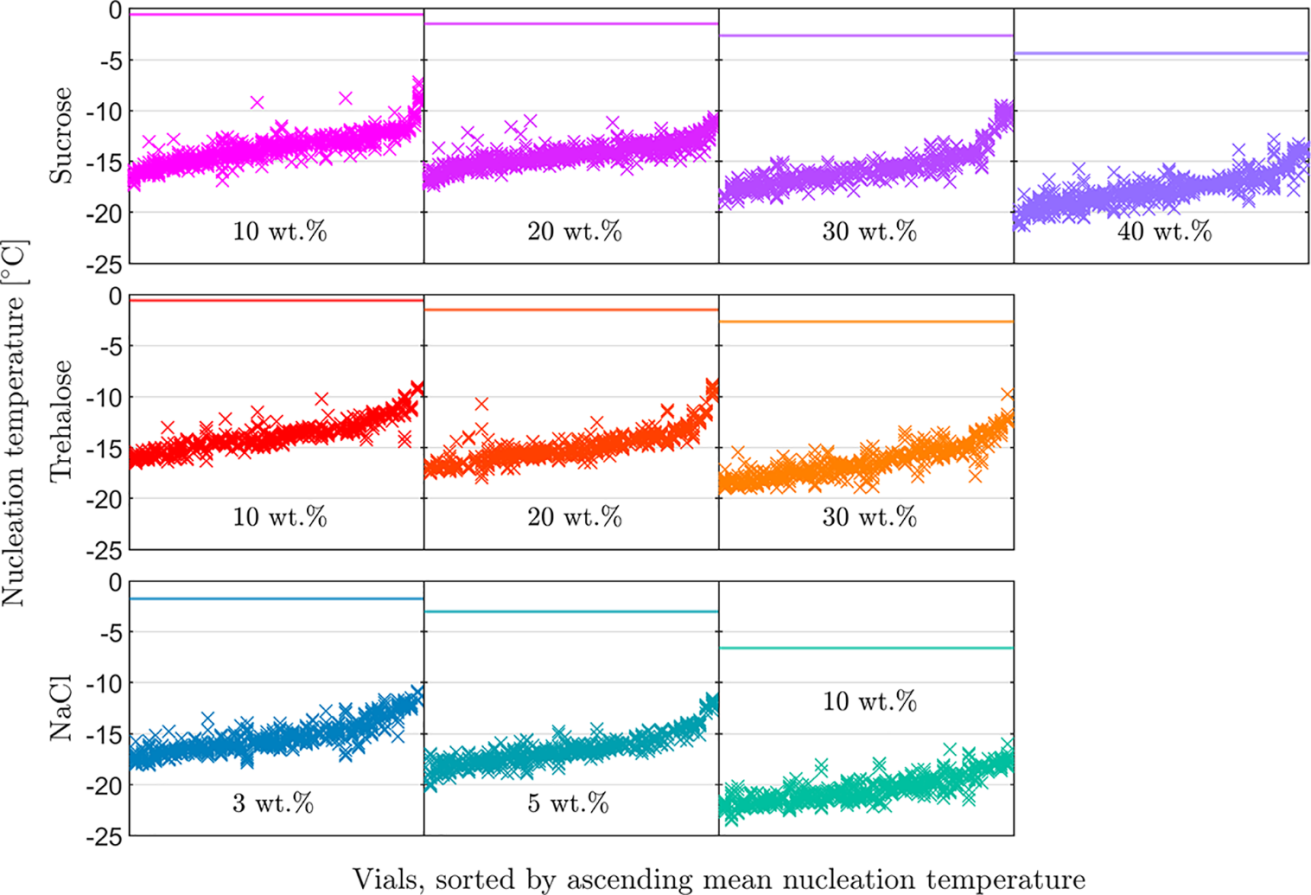
Overview of all measured nucleation temperatures. Each panel shows the data for one solution composition: the twelve nucleation temperatures measured per vial are arranged in columns sorted by ascending mean nucleation temperature. The horizontal lines indicate the equilibrium freezing temperature. Top row: sucrose. Center row: trehalose. Bottom row: sodium chloride.

Further, it is worth noting that for the same solute, increasing concentration shifts nucleation toward lower temperatures, which is consistent with the fact that the equilibrium freezing temperature *T*^eq^ of the solution also decreases with increasing solute concentration (see Figure 2).^34−36^

Figure 3a illustrates a nucleation experiment within the binary water–solute phase diagram, with coordinates for water activity, *a*_w_, and temperature, *T*. Water activity is a convenient quantity to express the driving force for nucleation because—though it depends on the solute concentration—its temperature-dependence is negligible.^20,23,29^ The black solid line denotes the solid–liquid equilibrium between ice and solution. It is defined through the Schröder–van Laar (SvL) equation, which gives the equilibrium water activity *a*_w_^eq^(*T*) as a function of temperature, or conversely, the equilibrium freezing temperature *T*^eq^(*a*_w_^0^) as a function of the solution’s water activity *a*_w_^0^:^37^
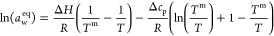
1where Δ*H* = 6002 J mol^–1^ is the latent heat of fusion of pure ice, Δ*c*_p_ = 38.03 J mol^–1^ K^–1^ is the difference in heat capacity between liquid water and ice, and *T*^m^ = 273.15 K is the freezing point of pure water, all evaluated at ambient pressure. Thus, with reference to Figure 3a, the cooling experiment starts from a point of coordinates (*a*_w_^0^, *T*^0^), and the solution state evolves along a vertical line whose points have coordinates (*a*_w_^0^, *T*). For *T* > *T*^eq^, the solution is in a thermodynamically stable state; for *T* < *T*^eq^ it enters a metastable state that persists until the phase transition is triggered by nucleation. The thermodynamic driving force for nucleation is the difference in chemical potential between ice and the solution, termed Δμ:
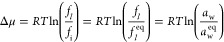
2where *f*_i_ and  are the fugacities of ice and of water in solution, respectively, and *f*_i_ equals the fugacity of water in solution when at equilibrium with ice crystals, i.e., . The last expression follows when considering that fugacity is the product of a reference fugacity and the activity of water. With reference to the schematic in Figure 3a, we note that the distance of the solution’s state to the equilibrium can be expressed either (i) as difference of the activities, i.e., Δ*a*_w_ = *a*_w_^0^ – *a*_w_^eq^(*T*), as typically used in the atmospheric sciences;^20,23,29^ or (ii) as difference of temperatures, i.e., Δ*T* = *T*^eq^ – *T*, which is also called degree of supercooling and is preferred in pharmaceutical applications due to its experimental accessibility.^12,15,17^ The expression for the chemical potential can be rewritten in terms of these quantities when taking the appropriate simplifications:
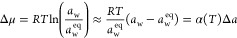
3
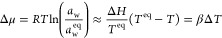
4As can be seen, interpreting Δμ as a function solely of Δ*a* requires (i) linearization of the logarithm and (ii) neglecting the temperature-dependency of the prefactor α. In contrast, to arrive at the expression for Δ*T*, no linearization is required; however, the Δ*c*_p_ term in the Schröder–van Laar equation is neglected.

**Figure 3:**
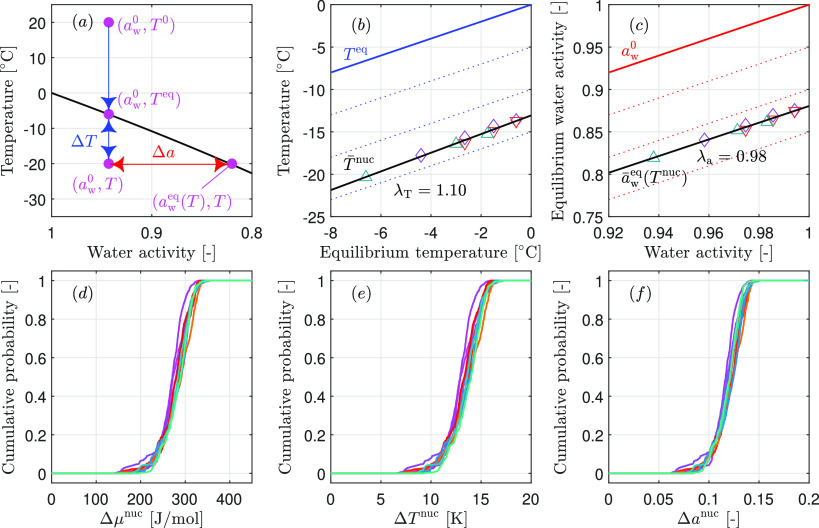
(a) Temperature–water activity diagram for the freezing process of aqueous solutions. The black line indicates the solution’s equilibrium properties, while the blue line shows the change in temperature during the process. (b and c) Mean nucleation temperatures *T̅*^nuc^ and mean nucleation activities *a̅*_w_^eq^(*T*^nuc^) for the ten studied solution compositions (colored markers). The data points are arranged by the equilibrium freezing point (blue) or by the corresponding water activity of the solution (red). The black line indicates the linear relationships between the solution’s equilibrium properties and the mean nucleation behavior. The dashed lines denote the isosupercooling (blue) or the isoactivity difference (red) curves. (d–f) Rescaled nucleation temperature distributions in terms of chemical potential difference Δμ^nuc^, of supercooling Δ*T*^nuc^, and of water activity difference Δ*a*^nuc^. The color-coding for the solution compositions is the same as that used in Figure 1.

Our working hypothesis is that Δμ, Δ*a*, and Δ*T* can all quantify the driving force for nucleation, even though likely with different accuracy. Moreover, we conjecture that the nucleation rate is given by a power law expression and that it can be calculated using either of the three, once the relevant parameters are estimated from experimental measurements:
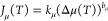
5
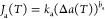
6
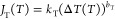
7where *J*_μ_, *J*_a_, and *J*_T_ denote the expected number of nucleation events per unit time and unit volume and *b*_μ_, *b*_a_, and *b*_T_ are constant exponents. The temperature-independent prefactors *k*_μ_, *k*_a_, and *k*_T_ are vial-specific constants with values that are log-norm distributed across vials; their negative decadic logarithm assumes a mean value of *a*_μ_, *a*_a_, or *a*_T_ and a standard deviation of *c*_μ_,*c*_a_, or *c*_T_, respectively. Such a distributed parameter is required to account for the experimentally observed variability in nucleation sites among vials.^5,10,19,21,38^ To keep the notation simple, subscripts for *J* and the associated kinetic parameters are used only when referring to a specific rate expression. While more complex rate expressions could be used to describe the nucleation kinetics, such as those based on the classical nucleation theory (CNT),^1,23,39^ we refrained from doing so: as we discuss below, all three power law expressions well describe the experimental data, so that more complex models would provide little benefit. To describe the variability in nucleation temperatures, we introduce the cumulative distribution function (CDF), *F*_*v*_(*t*), which denotes the probability that nucleation occurs in a specific vial *v* with fill volume *V* in the time interval [0, *t*]. The CDF is obtained by assuming that nucleation occurs through an inhomogeneous Poisson process (it would be homogeneous, if temperature were constant) in terms of the nucleation rate defined above, i.e., of the rate *J*(*T*):^4,11,40^
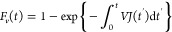
8The cumulative probability for nucleation to occur within the interval [0, *t*] for a *set of N vials*, termed *F*(*t*), is the mean of the CDFs of the individual vials:
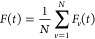
9When computing *F*(*t*), we consider the log-norm distribution of the prefactor *k* in the expressions for *J*(*T*): every vial *v* is assigned a unique value of *k*, namely −log_10_(*k*) = *a* + ξ_*v*_*c* with ξ_*v*_ = , whereby the probit function denotes the quantile function of the standard normal distribution.

The link among (i) the CDF *F*(*t*) as a function of time, (ii) the temperature-dependent nucleation rate *J*(*T*), and (iii) the distributions shown in Figure 1 follows from the nature of the freezing process: it is the temperature that changes over time during freezing, in our case in a predetermined manner, and it is this change that eventually triggers nucleation. To analyze whether Δμ, Δ*a*, and Δ*T* allow for an accurate quantitative description of nucleation, we follow a two-pronged approach: (i) first, the re-evaluation of the experimental results shown in Figure 1; (ii) second, the estimation of the model parameters in eqs 5–7, and the assessment of the quality of the fit thus obtained between experimental measurements and model results.

First, with reference to Figure 3a, we notice that when plotting the CDFs for the ten different solution compositions (originally shown in Figure 1) as a function of either Δμ^nuc^ in Figure 3d, Δ*T*^nuc^ in Figure 3e, or Δ*a*^nuc^ in Figure 3f, the ten curves overlap almost perfectly. They exhibit an average Δμ^nuc^ of 278 J mol^–1^, an average Δ*T*^nuc^ of 13.3 K, and an average Δ*a*^nuc^ of 0.119. This demonstrates that the three quantities indeed represent the driving force for nucleation, irrespective of the absolute temperature level, of the water activity (i.e., of solute concentration), and even of the nature of the solute. Furthermore, from the ten empirical cumulative distribution functions of the nucleation temperatures, one can calculate the average nucleation temperature and at that temperature the corresponding equilibrium water activity. These two quantities are plotted as a function of the equilibrium temperature (Figure 3b) and of the initial water activity (Figure 3c), respectively. In these panels, isolines of the driving force are plotted as blue and red dotted lines (the equilibrium isolines are solid). The black solid lines were obtained through linear regression of the experimental data (symbols). For the temperature-based driving force, the slope of the regression line is λ_T_ = 1.10, whereas it is λ_a_ = 0.98 in the case of the activity-based driving force. A slope of one would imply that the mean value of the driving force is independent of solution composition, so that the driving force quantitatively captures the effect of solution composition on nucleation. We hence conclude that both expressions accurately describe nucleation across the ten compositions investigated in this study and that the activity-based driving force is slightly more accurate than the temperature-based one. This is also suggested by the fact that the overlap of the cumulative distribution functions in Figure 3f is visually better than that in Figure 3e.

Second, by optimally fitting each empirical cumulative distribution with that calculated by eq 9 using the methodology developed recently,^19^ we have estimated the three model parameters, namely *a*, *b*, and *c*, in the three nucleation rate expressions (eqs 5–7). Their values are shown for all ten solution compositions in Figure 4 (symbols; the rows correspond to the three rate expressions, respectively), together with their confidence intervals (error bars, significance level α = 0.05, obtained by carrying out 500 Monte Carlo simulations each). In addition, we have estimated the nucleation parameters for the concatenated data set comprising all compositions; these parameter values are shown as thin black lines, with their confidence intervals given by the gray-shaded band. The cumulative distribution functions measured experimentally have been simulated with the estimated parameters and plotted as black solid lines in Figure 1 (where for the sake of clarity, only the lines calculated using *J*_T_ are shown, as those obtained using *J*_a_ and *J*_μ_ mostly overlap); the agreement is rather satisfactory, in both average nucleation temperature and shape of the distribution. When comparing the estimated parameter values for the three rate expressions in Figure 4, we notice that *b*_μ_ ≈ *b*_a_ ≈ *b*_T_ and *c*_μ_ ≈ *c*_a_ ≈ *c*_T_, whereas *a*_a_ ≪ *a*_T_ ≪ *a*_μ_. The similarity in the parameters *b* and *c* reflects the fact that they determine the shape of the nucleation temperature distributions, whereas parameter *a* acts as a scaling factor. Its order of magnitude is determined by the ratio between average values of the nucleation rate and of the driving force, the latter being very different when expressed as Δμ^nuc^, Δ*T*^nuc^, or Δ*a*^nuc^, since Δμ^nuc^ ≫ Δ*T*^nuc^ ≫ Δ*a*^nuc^. When comparing the estimated values across compositions, we do not observe any significant trend; the confidence intervals are similar in size in all cases, and for all ten solution compositions, they overlap with those of the concatenated set. These observations lead to the conclusion that all three descriptions are sufficiently accurate to describe nucleation from milliliter-scale solution and that a single set of parameters accurately quantifies the nucleation rate across solution compositions. This finding is of great interest to both researchers and practitioners in this field.

**Figure 4:**
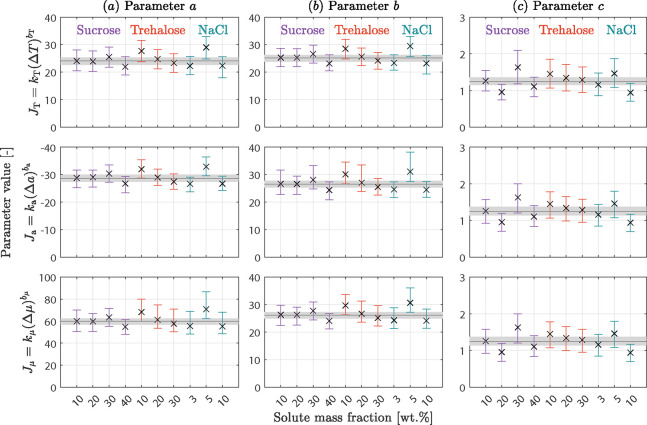
Estimated values for the three kinetic parameters *a*, *b*, and *c* as well as their confidence intervals (error bars) for the three expressions of the nucleation rate: temperature-based (top row), activity-based (center row), and chemical potential-based (bottom row). The black line surrounded by the gray region denotes the optimum parameter values and their confidence interval estimated from a concatenated data set comprising all ten solution compositions. The colors indicate the three different solutes used.

Let us contextualize this finding. Experimental studies on ice nucleation in small volumes have established that the effect of solution composition on mean nucleation temperature quantitatively matches the one on the solution’s water activity.^20,29−31^ Our study complements and extends this observation in multiple ways. First, we extend it to larger volumes at the milliliter-scale, where we generated an extensive data set of 6,000 nucleation events. Second, we show that not only water activity but also supercooling and chemical potential quantitatively describe the effect of solution composition on nucleation; for the chemical potential, to the best of our knowledge, this has not yet been shown. Concerning supercooling, the literature reports that the mean nucleation temperature decreases more strongly with solute concentration than the equilibrium freezing temperature (i.e., λ_T_ > 1).^30,41,42^ Our value of λ_T_ = 1.10 is smaller than most of those reported in the literature, which typically rely on experiments in microdroplets, but it quantitatively agrees with a recent study that considered milliliter-scale solutions.^8^ This suggests that the supercooling provides an accurate description of the nucleation rate in sufficiently large volumes; for smaller volumes, the use of either water activity or chemical potential may be more appropriate. Third, we underline that the nucleation rate is expressed as a power law of the thermodynamic driving force in this study. Alternative rate expressions such as those based on the classical nucleation theory, which consider additional kinetic effects, are commonly used in the literature.^5,23^ As our experimental data show, however, the rate is accurately quantified by considering thermodynamic effects only: more complex models are not required to describe ice nucleation at the milliliter-scale. Fourth and finally, we emphasize that the statistical analysis goes beyond assessing trends about the dependence of the mean nucleation temperature on solution composition: we explicitly studied whether there are significant effects of solution composition on the parameters in the nucleation rate expression. Despite carrying out large numbers of experiments and hence obtaining small confidence intervals, no significant differences were observed. The underlying modeling approach considers both the inherent stochasticity of nucleation (a Poisson process) and the variability in nucleation sites among vials (a log-norm distribution). This has been possible because the experimental data comprises multiple nucleation temperatures per vial, so that one can distinguish between the variability in nucleation temperatures within vials and the one among vials. To conclude, the analysis presented here provides a deeper understanding of ice nucleation and its controlling driving force, which can be of immediate value to the different fields where freezing processes play a major role.

## Methodology

The experimental methodology used to generate the nucleation temperature data reported in this contribution has been developed and explained in detail in our earlier work.^19^ Here, we provide a summary, focusing on those aspects that are relevant to this work. All experiments were carried out in a second-generation Crystal16 instrument (Technobis Crystallization Systems) that was customized to extend the attainable temperature range down to −35 °C. To ensure a sufficient cooling capacity, the instrument was connected to a thermostat (Huber unistat 430, set to −10 °C). For the solutions comprising 10 wt %, 20 wt % and 40 wt % sucrose, four experiments were conducted, and for the remaining ones three; the data for 20 wt % sucrose was already analyzed earlier (see Series 1–4 in Deck and Mazzotti^19^). Each experiment comprised 12 freeze–thaw cycles in 15 vials, amounting to a total of 540 or 720 nucleation events per solution composition, and a total of 495 monitored vials, i.e., 5940 nucleation events, across compositions.

All experiments were carried out using the same experimental protocol: during each cycle, the temperature was decreased from +20 °C to −25 °C with a constant cooling rate of 0.6 K min^–1^. The vials with 11.6 mm outer diameter were filled with 1 mL of aqueous solution using a micropipet (Socorex Acura 825). For each experiment, a fresh stock solution was prepared using deionized water (Millipore, Milli-Q Advantage A10 system) and solute. Sucrose (Sigma-Aldrich, BioXtra grade, >99.5% purity), trehalose (Sigma-Aldrich, dihydrate, from starch, >99% purity), and sodium chloride (Sigma-Aldrich, puriss. p.a. >99.5%) were used as solutes in this work. All stock solutions were filtered (0.22 μm hydrophilic PTFE syringe filter) before insertion into the glass vials (Lab Logistics Group GmbH, 1.5 mL). The time and temperature of nucleation in a vial were detected based on the rapid rise in temperature due to the fast crystal growth that follows nucleation. A thermocouple (K-type, Inconel 600, certified by Picolog, sampling interval 1s) was inserted into each vial for online temperature monitoring.

It is worth underlining two experimental challenges that are associated with the measurement of the nucleation kinetics. First, freezing experiments have to be carried out under well-controlled conditions to ensure that the experimentally observed variability in nucleation temperatures is indeed dominated by the stochastic nature of nucleation and not by experimental error. Doing so is challenging at all scales;^6,19^ the instrument we use here allows for highly automated, long-term freeze–thaw experiments. We achieve a temperature accuracy of about ±0.15 K that we consider sufficient compared to the width of the measured nucleation temperature distributions, which is on the order of 5–7 K. Second, the experimental data set must comprise nucleation temperatures both from a large number of vials and from a large number of freeze–thaw cycles: a single experiment hence comprises 12 freeze–thaw cycles in 15 vials, amounting to 180 nucleation events. Such a large data set is essential because different types of variabilities may be observed when considering the distributions of nucleation temperatures within vials, across vials, and across experiments; the stochastic description of ice nucleation has to account for this, as in the methodology we presented earlier^19^ and as it has been done with respect to microdroplets in the atmospheric sciences.^5,10,21^

To estimate the parameters in the rate expressions as well as their uncertainty, we have utilized the approach presented in our earlier work.^19^ When analyzing the thermal evolution curves of the 495 vials that were monitored, we encountered technical issues with the inserted thermocouples in four vials. Hence, these four vials were excluded from the data analysis (1 vial each for 3 wt % NaCl and 10 wt % sucrose, 2 for 40 wt % sucrose). The equilibrium freezing point data required for the computation of the supercooling has been obtained from experimental contributions in the literature for sodium chloride^36^ and for sucrose and trehalose.^35^ Relevant properties for all solution compositions are summarized in Table 1.

**Table 1:** Relevant Data for the Ten Selected Solution Compositions^a^

Solution composition	*T*^eq^ [°C]	*a*_w_^0^ [-]	Δ*T̅*^nuc^ [K]	Δ*a̅*^nuc^ [-]	Δ*μ̅*^nuc^ [J mol^–1^]
Sucrose, 10 wt %	–0.6	0.994	13.1	0.119	275
Sucrose, 20 wt %	–1.5	0.986	12.8	0.116	269
Sucrose, 30 wt %	–2.65	0.974	13.0	0.117	273
Sucrose, 40 wt %	–4.4	0.958	13.4	0.118	278
Trehalose, 10 wt %	–0.6	0.994	13.1	0.119	276
Trehalose, 20 wt %	–1.5	0.986	13.4	0.121	281
Trehalose, 30 wt %	–2.65	0.974	13.7	0.122	285
NaCl, 3 wt %	–1.75	0.983	13.5	0.121	283
NaCl, 5 wt %	–3.0	0.971	13.4	0.120	280
NaCl, 10 wt %	–6.6	0.938	13.7	0.119	284

a The equilibrium freezing temperatures *T*^eq^ were sourced from the literature, with a precision of 0.05 K.^35,36^ The corresponding water activity *a*_w_^0^(*T*^eq^) was computed through eq 1. The mean values of the thermodynamic driving force, namely, Δ*T̅*^nuc^, Δ*a̅*^nuc^, and Δ*μ̅*^nuc^, were computed from the experimental data set generated in this work.
